# Postural Sway in Parkinson's Disease and Multiple Sclerosis Patients During Tasks With Different Complexity

**DOI:** 10.3389/fneur.2022.857406

**Published:** 2022-03-29

**Authors:** Elke Warmerdam, Maike Schumacher, Thorben Beyer, Patrik Theodor Nerdal, Linda Schebesta, Klarissa H. Stürner, Kirsten E. Zeuner, Clint Hansen, Walter Maetzler

**Affiliations:** ^1^Department of Neurology, Kiel University, Kiel, Germany; ^2^Innovative Implant Development (Fracture Healing), Division of Surgery, Saarland University, Homburg, Germany

**Keywords:** balance, inertial measurement unit, postural stability, static sway, task complexity

## Abstract

Neurological diseases are associated with static postural instability. Differences in postural sway between neurological diseases could include “conceptual” information about how certain symptoms affect static postural stability. This information might have the potential to become a helpful aid during the process of finding the most appropriate treatment and training program. Therefore, this study investigated static postural sway performance of Parkinson's disease (PD) and multiple sclerosis (MS) patients, as well as of a cohort of healthy adults. Three increasingly difficult static postural tasks were performed, in order to determine whether the postural strategies of the two disease groups differ in response to the increased complexity of the balance task. Participants had to perform three stance tasks (side-by-side, semi-tandem and tandem stance) and maintain these positions for 10 s. Seven static sway parameters were extracted from an inertial measurement unit that participants wore on the lower back. Data of 47 healthy adults, 14 PD patients and 8 MS patients were analyzed. Both healthy adults and MS patients showed a substantial increase in several static sway parameters with increasingly complex stance tasks, whereas PD patients did not. In the MS patients, the observed substantial change was driven by large increases from semi-tandem and tandem stance. This study revealed differences in static sway adaptations between PD and MS patients to increasingly complex stance tasks. Therefore, PD and MS patients might require different training programs to improve their static postural stability. Moreover, this study indicates, at least indirectly, that rigidity/bradykinesia and spasticity lead to different adaptive processes in static sway.

## Introduction

Static postural stability is an important contributor to the balance control framework. Effective static postural stability performance depends on an intense interplay of sensory information that is processed by balance control filters and responding motor commands (1). Multiple systems (visual, vestibular, somatosensory) have to interact with each other to maintain balance (2). To maintain a state of equilibrium during quiet stance, the center of mass (COM) of the body needs to be above the base of support (BOS), which is defined by the position of the feet (3). Movements of the COM during quiet stance are used as a measure for static postural stability. A wearable sensor, worn on the lower back, can provide indirect information on even small movements of the COM. Inertial measurement units (IMUs) have shown to be a valid and reliable tool to quantify static postural sway (4, 5), although the extractable variables show markedly different reliabilities (6). Sway parameters that can be extracted with IMUs are e.g., area, velocity, acceleration and jerk (quick compensatory movements) (7).

It has been shown that static postural stability is often compromised with advancing age (8, 9), reflected, e.g., by greater postural sway of older adults compared to young adults (10, 11). Concerning specific assessment of side-by-side stance, healthy older adults showed significantly greater medio-lateral (ML) sway, during semi-tandem stance greater anterio-posterior (AP) and ML sway and during tandem stance greater AP sway compared to young controls (12). Postural sway can also be used as an indicator of fall risks in the aging population as sway area is associated with falls in older adults (13, 14). These age-related changes are most probably caused by anatomical and physiological alterations leading to impairments of the somatosensory system (9) and the vestibular system (15), and also by age-related changes of the multisensory integration of the central nervous system (16, 17).

Neurological diseases, such as Parkinson's disease (PD), are often associated with static postural instability. The postural reflexes to maintain stability are altered in PD patients compared to healthy adults. PD patients show a disruption of the precisely coordinated execution of agonist and antagonist muscles (associated with bradykinesia and rigidity), which results in difficulty to maintain static postural stability (18–20). Moreover, PD patients often have limb and axial rigidity (stiffness). While this stiffening reduces the area of sway (21), it does prevent the use of flexible responses, thereby aggravating the deterioration of postural reflexes (22, 23). PD patients with postural instability and gait difficulty are more likely to suffer falls compared to PD patients without postural instability or gait difficulty (24). It has been shown that PD patients show higher sway acceleration, jerk and sway velocity during upright standing position compared to age-matched healthy controls (25, 26). It has also been shown that PD patients have increased jerkiness during the performance of cognitive task (27), suggesting an interaction of higher cognitive functions and multisensory integration with static balance mechanisms.

A neuroinflammatory disease affecting static postural stability is multiple sclerosis (MS). MS is a chronic inflammatory disease that leads to demyelination and neurodegeneration in the central nervous system (28). The reduced static postural stability is most probably due to lower-limb spasticity, although reduced sensation in the feet, slowed spinal somatosensory conduction, and a delay in sensory and motor integration may also contribute (29). Spasticity is described as velocity-dependent increase in muscle tone (30) and affects about 80% of people with MS (31). MS patients could be differentiated from healthy controls by ML sway path length and ML range of sway acceleration amplitude during upright stance on a foam surface (32). Even when compared to patients with other neurological diseases, such as PD and stroke, larger sway area and larger sway in AP and ML direction during upright stance on a foam surface in eyes open and eyes closed conditions could be found in MS patients (33). Static sway parameters in MS patients are also associated with increased fall risk (34).

Based on existing information, static postural stability measures differ between healthy adults and patients with PD or MS (27, 35). However, there is limited information on how static postural stability differs between two different neurological diseases (e.g., PD and MS), which could also include “conceptual” information of about, e.g., how rigidity and bradykinesia (predominant in PD) and spasticity (predominant in MS) affect static postural stability. Moreover, it is unclear whether healthy adults, PD and MS patients adapt differently to an increasing difficulty of stance tasks. This information might have the potential to become a helpful aid during the process of finding the most appropriate treatment and training program, and understand the adaptation strategies of the body as a consequence of different neurological symptoms.

Therefore, this study investigated static postural sway performance with IMUs of PD and MS patients, as well as of a cohort of healthy adults using a protocol with three increasingly difficult static postural tasks, in order to determine whether the postural strategies of the two disease groups differ in response to the increased complexity of the balance task.

## Materials and Methods

### Study Participants

We assessed 56 healthy adults, 24 individuals with PD on medication and 11 individuals with MS. Healthy adults were recruited via flyers in public facilities. The patients were recruited from the wards of the neurology department of the University hospital of Schleswig-Holstein (UKSH), Campus Kiel, Germany. Exclusion criteria were a Montreal Cognitive Assessment (MoCA) test score below 15 and the need of a walking aid. All participants underwent a clinical examination and a static postural stability assessment (see below). The clinical examination consisted of the motor part of the Unified Parkinson's Disease Rating Scale revised by the Movement Disorders Society (MDS-UPDRS III) and the MoCA. The MS patients were additionally evaluated with the Expanded Disability Status Scale (EDSS). The full study protocol can be found elsewhere (36).

The ethical board of the medical faculty of Kiel University approved this study (project number D438/18) and all participants gave their written informed consent.

### Postural Stability Assessment

**Figure 1:**
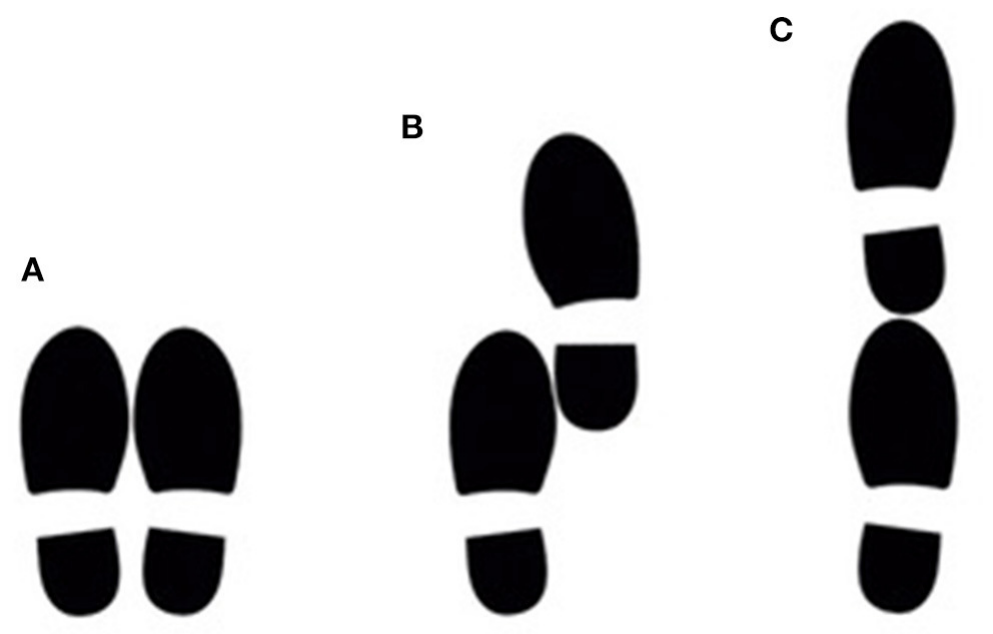
The position of the feet during the side-by-side stance **(A)**, semi-tandem stance **(B)** and tandem stance **(C)**. Participants had to hold this position for 10 s.

The study participants performed the three static postural stability tasks that are included in the short physical performance battery (37). For the first task, participants were asked to stand with their feet together, side-by-side. For the second task, they were asked to stand with the side of the heel of one foot touching the big toe of the other foot, the semi-tandem position. For the third task, they were asked to stand with the heel of one foot in front of the toes of the other foot, the tandem position (Figure 1). The participants were allowed to put either foot in front (for the semi-tandem and tandem position) and were asked to stand still and hold each position for 10 s.

### Instrumentation and Data Analysis

Postural sway was measured using an IMU (MyoMotion, Noraxon Inc, Scottsdale Arizona, USA) worn on the lower back. The parameters were extracted from the acceleration data as described and validated in another study (7). An explanation of the parameters can be found in Table 1. The acceleration, velocity and jerk parameters were calculated in both AP and ML direction.

**Table 1:** Description of the parameters used in this study.

**Parameter**	**Explanation**
Area [m^2^/s^4^]	The area covered by the sway
Velocity [m/s]	Root mean square of the velocity time series (obtained by integrating the acceleration)
Acceleration [m/s^2^]	Root mean square of the acceleration time series
Jerk [m/s^3^]	Root mean square of the jerk time series (time derivative of acceleration), providing a measure of quick compensatory movements

### Statistical Analysis

ANOVAs were used to analyze the differences between the groups per balance task. Homogeneity was corrected according to Welch and *Post Hoc* testing was performed according to Tukey's method. Repeated measures ANOVAs were used to analyze the differences between balance tasks within each group. If the assumption of sphericity was rejected, a Greenhouse-Geisser correction was used. *Post Hoc* testing was performed with Holm's correction.

**Figure 2:**
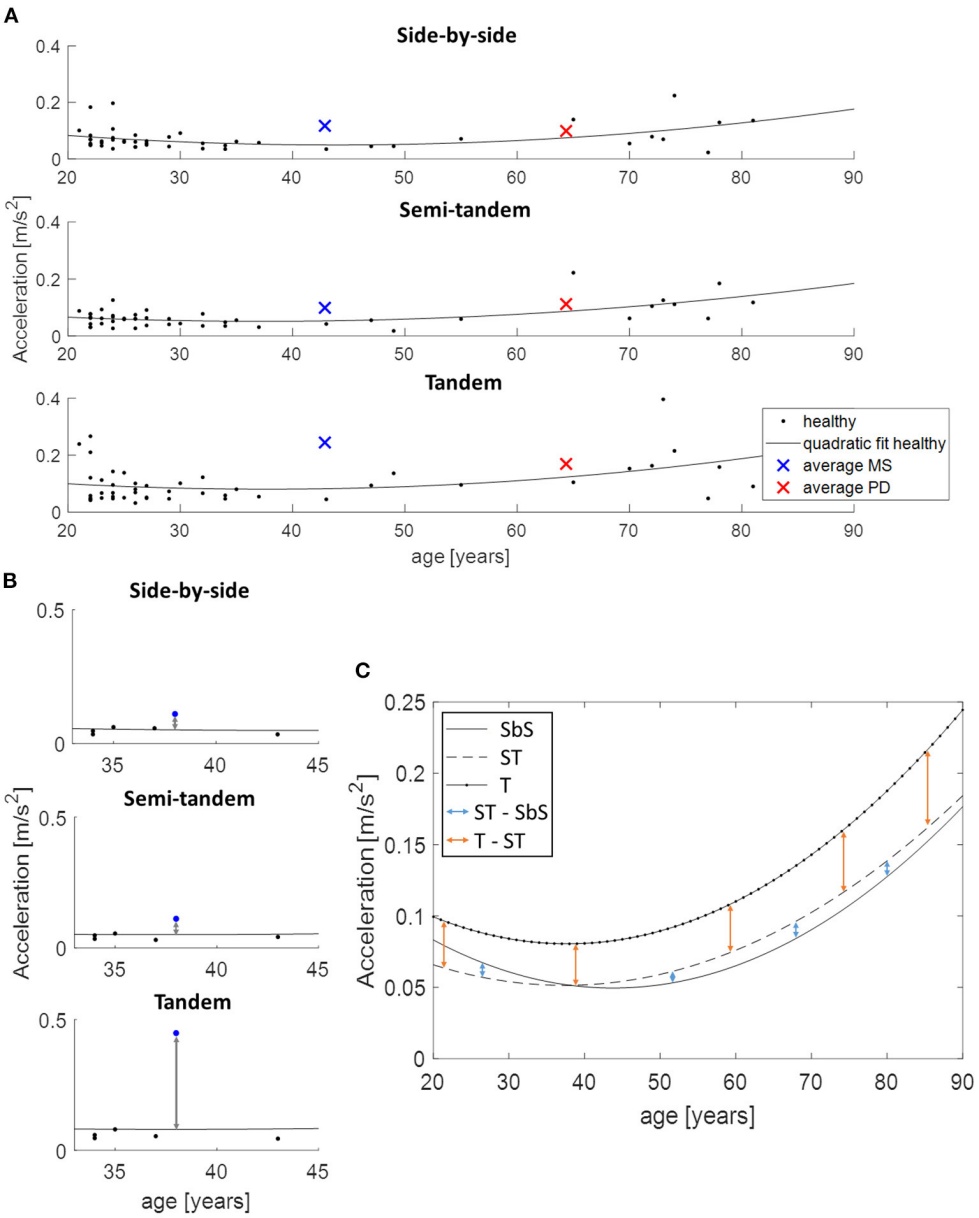
Use of the quadratic function to allow age-corrected comparisons between disease groups, demonstrated on acceleration in medio-lateral direction. **(A)** An example of the quadratic fit based on the data of all healthy adults and the average values of the multiple sclerosis (MS, blue cross) and Parkinson's disease (PD, red cross) patients. **(B)** The distance between the performance of an MS patient (blue dot) and the estimated value of the age-matched healthy adults is presented. These distances are subtracted from each other to calculate the change between the tasks. **(C)** Shows the calculation of the changes between the tasks for the healthy adults, where the quadratic fits are subtracted from each other. SbS, side-by-side stance; ST, semi-tandem stance; T, tandem stance.

Directly comparing the PD and MS group was not possible because of the mean age difference of 21 years. Therefore, the data of the healthy participants was used to estimate what the value of the parameters would be at each age with the use of a quadratic function (Figure 2A). For each patient and each task, the distance between the quadratic fit and their value at the appropriate age was calculated (Figure 2B). These obtained values were then used to calculate the change between the tasks for both PD and MS patients by subtracting semi-tandem stance from side-by-side stance and by subtracting tandem stance from semi-tandem stance. To be able to see how these changes relate to healthy adults, the average differences between the quadratic fits were calculated (Figure 2C).

## Results

In total 91 participants were measured, however not all participants were able to perform all three balance tasks (Table 2). Only the 69 participants that were able to perform all three tasks were included in the analysis (Table 3). The included PD patients had a Hoehn & Yahr score between 1 and 3, and the MS patients had a EDSS between 1 and 4. This indicates that the patients had mild to moderate disease severity with limited disability(38, 39).

**Table 2:** Number of participants that were able to perform the stance tasks.

	**Side-by-side**	**Semi-tandem**	**Tandem**
	**stance**	**stance**	**stance**
Healthy adults	56	55 (98%)	47 (84%)
Parkinson's disease	23	20 (87 %)	14 (61%)
Multiple sclerosis	12	11 (92%)	8 (67%)
All participants	91	86 (95%)	69 (76%)

**Table 3:** Demographics and clinical scores of the participants that were able to perform all three balance tasks.

	**Healthy**	**Parkinson's**	**Multiple**
	**adults**	**disease**	**sclerosis**
Female/male	22/25	4/10	4/4
Age [years]	37 ± 19	64 ± 10	43 ± 13
Weight [kg]	74 ± 12	87 ± 17	77 ± 6
Height [m]	1.78 ± 9	1.77 ± 9	1.80 ± 8
Disease duration [years]		8 ± 6	7 ± 9
MDS-UPDRS score (0–132)	2 ± 3	28 ± 20	7 ± 6
MoCA score (0–30)	28 ± 2	24 ± 2	28 ± 1
EDSS score (0–10)			2 ± 1
Patients with one or more falls in the last 12 months (%)		64	50

*Data are presented as mean ± standard deviation, or as numbers (female/male). EDSS, Expanded Disability Status Scale; MDS-UPDRS III, third part of the Unified Parkinson's Disease Rating Scale revised by the Movement Disorder Society; MoCA, Montreal Cognitive Assessment*.

### Comparison of Static Sway Performance Between Groups Within One Balance Task

**Figure 3:**
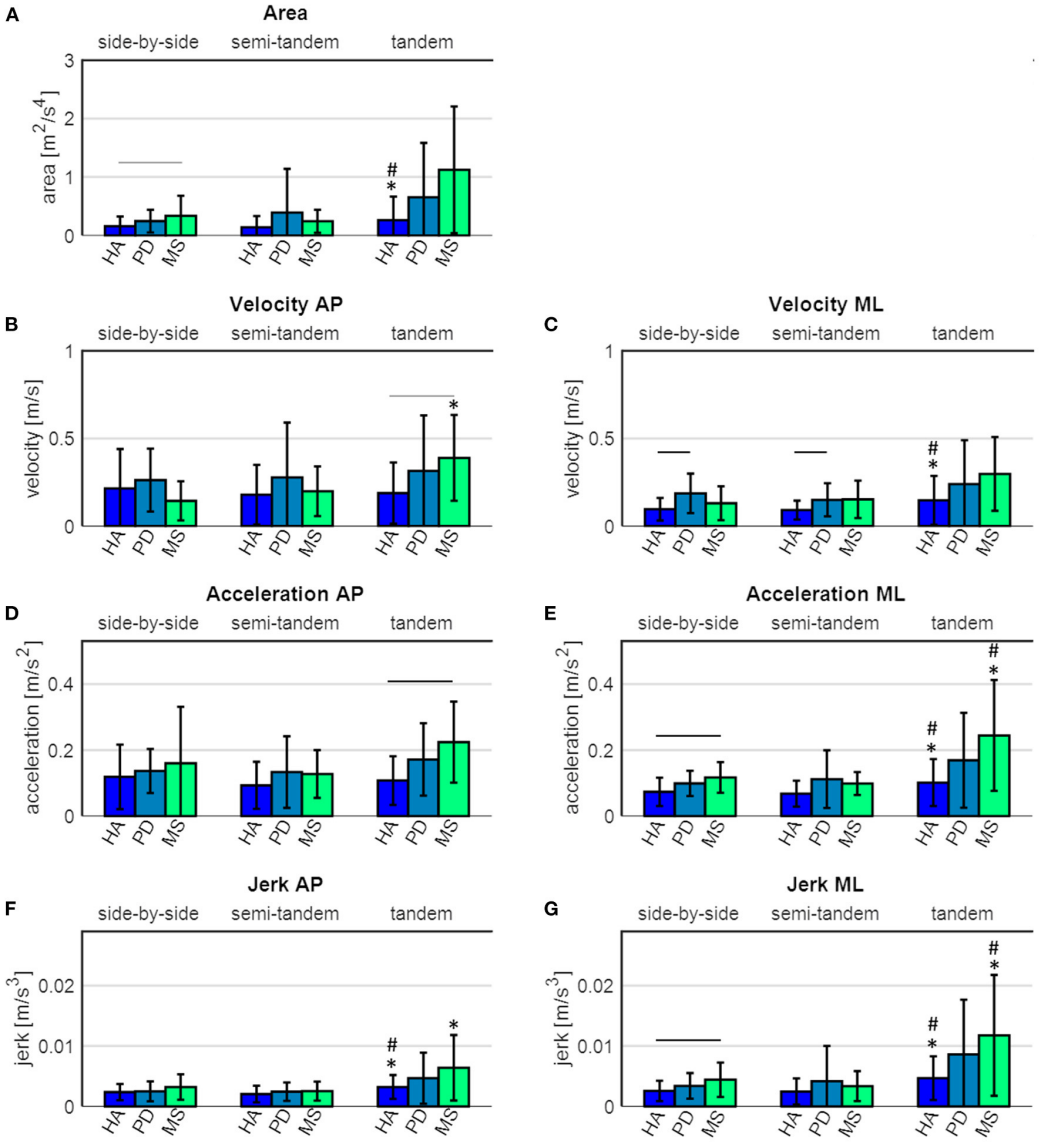
Sway parameters of healthy adults (HA), Parkinson's disease (PD) and multiple sclerosis (MS) patients. **(A)** sway area, **(B)** velocity in antero-posterior (AP) direction, **(C)** velocity in medio-lateral (ML) direction, **(D)** acceleration in AP direction, **(E)** acceleration in ML direction, **(F)** jerk in AP direction and **(G)** jerk in ML direction. The black horizontal lines indicate a significant difference between the groups, the grey horizontal lines indicate a tendency toward a difference between the groups. * indicates a significant difference compared to the side-by-side stance, # indicates a significant difference compared to the semi-tandem stance.

During **side-by-side stance**, significant differences between the groups were found for sway area (*p* = 0.042), velocity in ML direction (*p* = 0.035), acceleration in ML direction (*p* = 0.011) and jerk in ML direction (*p* = 0.034). Post hoc tests revealed that PD patients showed significantly higher velocity in ML direction (*p* = 0.001), compared to healthy adults (Figure 3). MS patients showed a significantly higher acceleration (*p* = 0.023) and jerk (*p* = 0.040) in ML direction, and a tendency toward a larger sway area (*p* = 0.055), compared to the healthy adults (Figure 3).

During **semi-tandem stance**, significant differences between the groups were found for velocity in ML direction (*p* = 0.020). *Post hoc* tests revealed that PD patients had a significantly higher sway velocity in ML direction (*p* = 0.049), compared to the healthy adults. During **tandem stance**, significant differences between the groups were found for velocity (*p* = 0.026) and acceleration (*p* = 0.005) in AP direction. *Post hoc* tests revealed that the MS patients showed a significantly higher acceleration in AP direction (*p* = 0.009) and a tendency toward a higher velocity in AP direction (*p* = 0.053), compared to the healthy adults (Figure 3).

There were no significant differences between PD and MS patients in any of the three tasks (Figure 3).

### Comparison of Static Sway Performance Within One Group Between Different Tasks

Healthy adults showed a significant increase in sway area (*p* = 0.044), velocity in ML direction (*p* = 0.007), acceleration in ML direction (*p* = 0.001) and jerk in both AP (*p* = 0.001) and ML (*p* = 0.001) direction between the side-by-side and tandem stance (Figure 3 and Table 4). The sway area (*p* = 0.028), velocity in ML direction (*p* = 0.007), acceleration in ML direction (*p* < 0.001) and jerk in both AP (*p* <0.001) and ML (*p* < 0.001) direction significantly increased between the semi-tandem and tandem stance (Figure 3).

**Table 4:** Significant changes of sway parameters between tasks.

	**HA**		**PD**		**MS**	
	**SbS-ST**	**ST-T**	**SbS-ST**	**ST-T**	**SbS-ST**	**ST-T**
Area						
Velocity AP						
Velocity ML						
Acceleration AP						
Acceleration ML						
Jerk AP						
Jerk ML						

*The black squares indicate a significant difference between two tasks per participant group. AP, antero-posterior; HA, healthy adults; ML, medio-lateral; MS, multiple sclerosis; PD, Parkinson's disease; SbS, side-by-side stance; ST, semi-tandem stance; T, tandem stance*.

MS patients showed a significant increase in sway velocity (*p* = 0.032), acceleration in ML direction (*p* = 0.023) and jerk in ML (*p* = 0.025) and AP (*p* = 0.047) direction between side-by-side and tandem stance. Moreover, they showed a significant increase in sway acceleration in ML direction (*p* = 0.015) and jerk in ML direction (*p* = 0.017) between semi-tandem and tandem stance (Figure 3).

PD patients did not show a significant change in any of the sway parameters between the tasks (Figure 3).

### Comparison of Static Sway Adaptations With Increasing Task Complexity Between PD and MS Patients

**Figure 4:**
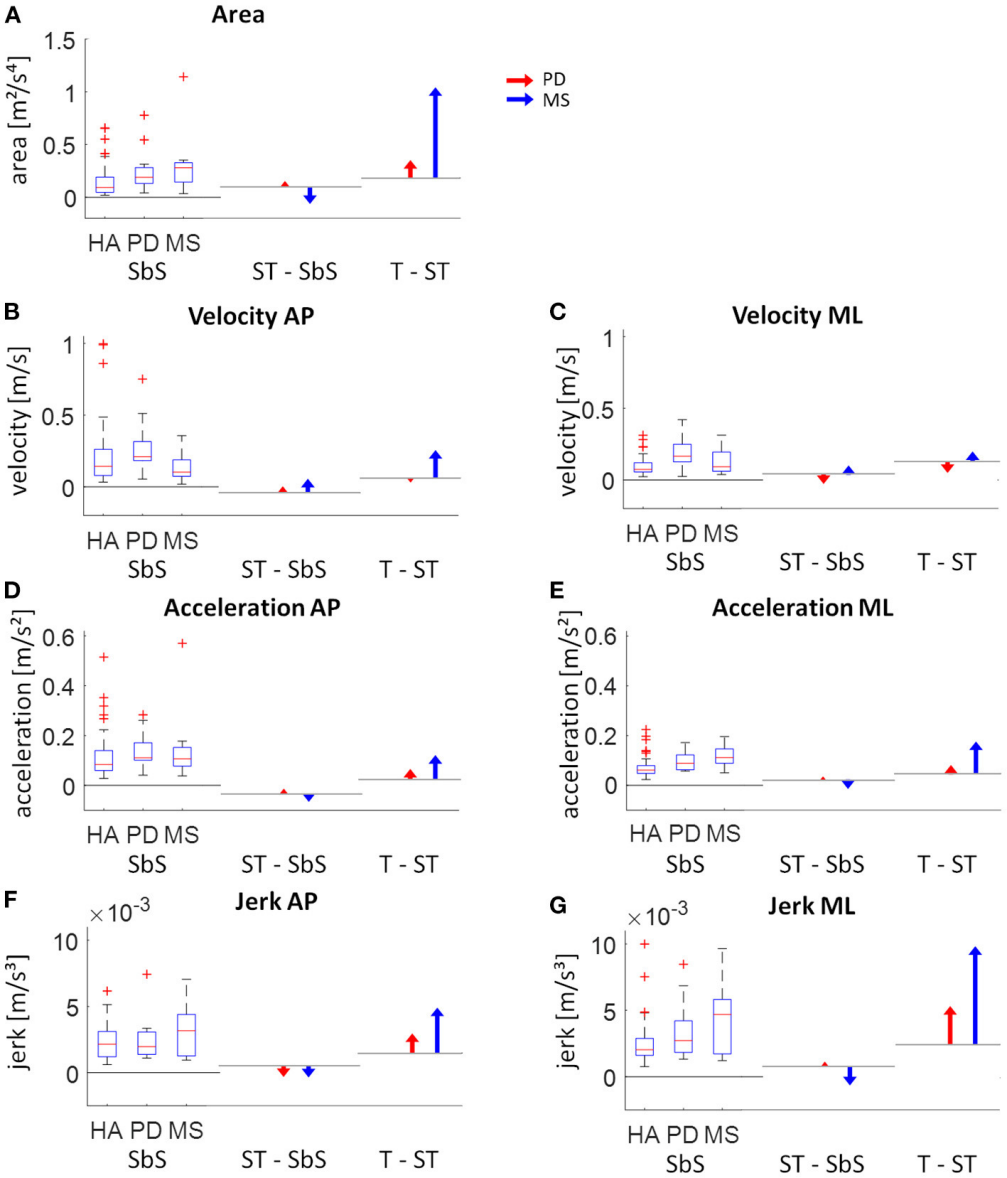
Visual presentation of the intra-group changes of respective static sway parameters. The left part of each graph shows the absolute values for side-by-side (SbS) stance (with boxplots showing the median, upper and lower quartiles, the 1.5 interquartile range and the outliers). The middle and right part of each graph show the age-corrected differences relative to the healthy adults (HA) between SbS and semi-tandem stance (ST - SbS) and between ST and tandem stance (T - ST) for the Parkinson patients (PD; red) and multiple sclerosis patients (MS; blue). The horizontal lines indicate the change in sway for the healthy adults between the tasks (calculated by subtracting the quadratic fits from each assessment). **(A)** sway area, **(B)** velocity in antero-posterior (AP) direction, **(C)** velocity in medio-lateral (ML) direction, **(D)** acceleration in AP direction, **(E)** acceleration in ML direction, **(F)** jerk in AP direction and **(G)** jerk in ML direction.

Clear differences in adaptations to increasingly complex stance tasks between PD and MS patients were observed (Figure 4). PD patients showed gradual increases in sway parameters from the least to the most complex stance task. MS patients showed a tendency toward smaller values compared to the healthy adults in sway parameters from the side-by-side stance to the semi-tandem stance, except for velocity. Velocity in MS patients was relatively low during side-by-side stance, compared to the other groups and showed small increases from the side-by-side stance to the semi-tandem stance. MS patients showed large increases in sway between the semi-tandem and tandem stance. This increase was for the area and jerk in ML direction even more than two times the values of the side-by-side stance (Figure 4).

## Discussion

This study investigated static postural sway performance of PD and MS patients with three increasingly difficult static postural tasks. The aim of the study was to analyze the (1) postural sway performance and (2) postural adaptation strategies to increasingly difficult tasks of the PD and MS patients, to evaluate whether different pathologies of the nervous system lead to distinct postural sway behavior under increasingly challenging conditions. Our results were able to confirm findings of existing studies measuring static postural sway of healthy adults, PD and MS patients. To our knowledge this is the first study that not only compared the sway performances of PD and MS patients to healthy controls and directly to each other, but also corrected for age differences between the patient groups for a fair comparison and looked at the change in sway performance with tasks of increasing difficulty. The motivation for this study is to gain a better understanding of the influence of bradykinesia/rigidity and spasticity on static balance, and to derive from the findings potentially more specific therapies for those affected, e.g., with a history of falls caused by the respective pathologies.

### Comparison of Static Sway Performance Between Groups Within One Balance Task

The only statistically significant differences between groups could be found when the groups were compared to healthy adults. In line with a previous study that compared PD patients to age-matched healthy adults, we found higher sway velocities of PD patients (26) during the side-by-side stance.

Unlike another study, at our study the larger sway areas and higher jerk of PD patients compared to healthy controls were not significant (25). However, in the previous study participants were untreated and had to stand still for 120 s. In the present study, only PD patients on dopaminergic medication as well as patients with a combination of deep brain stimulation and dopaminergic medication were included. Thus, it could be that dopaminergic medication as well as deep brain stimulation lead to at least some improvements in static postural sway. There are however discordant findings about this effect in the literature (40–43). MS patients showed a higher sway area, acceleration (ML direction) and jerk (ML direction) during side-by-side stance compared to healthy adults, which is comparable with other studies (32, 44).

During the semi-tandem stance there was only a significant difference between healthy adults and PD patients for the velocity in ML direction. The differences that were found between healthy adults and MS patients during the side-by-side stance are not present anymore during the semi-tandem stance. It seems that the MS patients have “decreased” their sway during this slightly more complex task. It could thus be that, in MS, the increase in the base of support in AP direction leads to a more stable static balance.

During the tandem stance, only the velocity and acceleration in AP direction were significantly different between the healthy adults and MS patients. The difficulty with the tandem stance in MS patients could be caused by spasticity. It has been shown that spasticity negatively affects balance in patients with MS, even with low levels of spasticity (45). MS patients with higher levels of spasticity have also been described to have more postural sway in ML direction (46), which is basically in accordance with our findings.

As a general comment, these differences across studies may be due to different (sub)cohorts included in respective studies. However, since the current study does not focus on this comparison but mainly on differences between sway tasks (see below), we do not consider these differences relevant here.

### Comparison of Static Sway Performance Within One Group Between Different Tasks

Adaptation of the body posture is required to compensate for the change in the base of support with the increasingly difficult stance tasks. Differences in these adaptation strategies between groups were visible. Both healthy adults and MS patients showed changes in sway parameters. These results suggest that the healthy adults and MS patients seem to be able to adapt their postural sway to the complexity of the task. However, only the healthy adults were able to increase area significantly over increasingly difficult balance tasks, which could at least indirectly speak for the possibility that both MS and PD already sway so much in the simple side-by-side task that an adequate increase of the sway area is no longer possible even in more difficult tasks.

PD patients did not show a significant increase in postural sway parameters with increasing complexity of the task and seem therefore not able to adapt their sway adequately to increasingly difficult balance tasks, or are not able to afford an increase in postural sway without exceeding their limits of stability. This could be related to PD symptoms, but could also be due to the age-related impairments of the somatosensory (9) and the vestibular system (15), making flexible and quick postural responses more difficult.

### Different Pathologies Are Associated With Different Postural Sway Behavior Under Increasingly Complex Conditions

Our study shows clear differences in postural adaptation strategies between PD and MS patients. PD patients do not seem to adapt more to increasingly difficult static balance tasks than the healthy adults, although PD patients have lower postural stability and increased fall risk compared to age-matched healthy adults (19). It could be that the PD patients are not able to adapt their sway much because of their bradykinesia and rigidity. However, it is also known that rigidity can increase postural sway by preventing the use of flexible responses and thereby aggravating the deterioration of postural reflexes (22, 23). MS patients increased their sway a lot, especially area, acceleration and jerk, when adapting to the most complex task. It is known that more than 80% of the MS patients have spasticity (31, 47) and that spasticity has a negative effect on postural stability (45, 46). When we consider the results of our study and those of another study (48) together, it seems likely that MS patients have substantial sway problems especially when the base of support is narrow (or the ML dimension small), such as during the tandem stance. A timed 3 m tandem walk was also significantly better in separating asymptomatic and symptomatic (in the motor or cerebellar functional system) MS patients compared to a timed 25-foot walk (49).

We suggest that whether the patients can perform control-like (PD patients) or not (MS patients) could have to do simply with the location of the pathology within the central nervous system. In PD, mainly the basal ganglia are affected. Due to the localization of the affected areas in the parenchymal structures and large networks of the central nervous system, there are extensive possibilities for compensation, both by upstream and downstream nodes (50), and by using alternative pathways or even networks (51). In MS patients the involvement of the pyramidal tract explains spasticity. The pyramidal tract is markedly different from the basal ganglia. This tract is the brain's only gateway to motor movement, and if this gateway is damaged, then no compensation can occur. This is well-studied, for example, in paraplegic syndromes (52). This idea is supported by results from exercise training on falls in these diseases. Whereas, PD patients are obviously able to reduce the number of falls after specific trainings (53–55), exercise studies suggest that this may not be the case in MS (55, 56).

Based on the results of this study, the following may be stated with regard to therapies: PD patients should benefit from exercises that are also effective for adults without PD. This is supported by the “control-like” adaptation of sway to increasingly difficult balance tasks. In addition, they should be trained to fully exploit their sway potential. Our data suggest that PD patients tend to underuse the whole arsenal of adaptation possibilities (no significant increase from easy to difficult task exercise). For this type of training, there is certainly a lesson to be learned from the Lee Silverman Treatment Strategy (57), although specific studies in this field are still lacking. In MS, due to the presumed lack of compensation possibilities at the level of the damaged pyramidal tract, preventive training seems more appropriate. Recent studies showed for example that Vojta reflex therapy seems to be improving balance in MS patients (58, 59).

### Strengths and Limitations of the Study

While most studies measured PD patients OFF medication, the present study included PD patients that were ON medication as well as patients with a combination of deep brain stimulation and medication. Both treatment options have been shown to influence postural sway performance (40–43). Some studies even suggest contradictory effects of dopaminergic medication and deep brain stimulation on postural sway (60). Therefore, putting all PD patients in one group could have had some effect on the results. However, by measuring PD patients ON medication and deep brain stimulation, this study provides more realistic information on the postural sway of PD patients in their daily lives.

The PD patients had a slightly lower general cognitive performance based on the MoCA score compared to the healthy adults and MS patients. In the PD patients the MoCA score is negatively correlated with the sway path length (61). Therefore, the lower cognitive performance in PD could have led to slightly higher sway values. Since we are basically presenting an intra-individual comparison of static balance across different difficulty levels, we do not assume that the preceding information is relevant to this study. Moreover, number of patients were relatively small and the day-to-day variability in the static sway parameters can be relatively high in neurological patients (6), therefore the results need to be interpreted with caution especially concerning sensitivity. Vice versa, it is very likely that the detected significances actually represent true differences.

## Conclusions

In this small sample of PD and MS patients, there were differences in postural adaptation strategies between PD and MS patients with increasingly complex static balance tasks. PD patients performed relatively control-like, whereas MS patients showed substantial changes between the increasingly complex tasks, especially between the two most complex tasks. These different postural adaptation strategies could be because of the disease-specific symptoms as well as the location of the main pathology in the central nervous system. Therefore, PD and MS patients might require different therapies to improve their static postural stability.

## Data Availability Statement

The raw data supporting the conclusions of this article will be made available by the authors, without undue reservation.

## Ethics Statement

The studies involving human participants were reviewed and approved by Ethical board of the medical faculty of Kiel University. The patients/participants provided their written informed consent to participate in this study.

## Author Contributions

EW, MS, and WM: concept and design of the study. EW, MS, TB, PN, LS, and WM: data acquisition and analysis. EW, MS, TB, PN, LS, KS, KZ, CH, and WM: interpretation of the results. All authors discussed the results and contributed to the final manuscript.

## Funding

We acknowledge financial support by Land Schleswig-Holstein within the funding programme Open Access Publikationsfonds.

## Conflict of Interest

The authors declare that the research was conducted in the absence of any commercial or financial relationships that could be construed as a potential conflict of interest.

## Publisher's Note

All claims expressed in this article are solely those of the authors and do not necessarily represent those of their affiliated organizations, or those of the publisher, the editors and the reviewers. Any product that may be evaluated in this article, or claim that may be made by its manufacturer, is not guaranteed or endorsed by the publisher.
